# Different functional roles for K^+^ channel subtypes in regulating small intestinal glucose and ion transport

**DOI:** 10.1242/bio.042200

**Published:** 2019-06-26

**Authors:** Chao Du, Siyuan Chen, Hanxing Wan, Lihong Chen, Lingyu Li, Hong Guo, Biguang Tuo, Hui Dong

**Affiliations:** 1 Department of Gastroenterology, Xinqiao Hospital, Third Military Medical University, Chongqing 400037, China; 2 Department of Gastroenterology and Hepatology, Chengdu Military General Hospital, Sichuan Province, Chengdu 610000, China; 3 Department of Gastroenterology, Affiliated Hospital, Zunyi Medical College, and Digestive Disease Institute of Guizhou Province, Zunyi 563003, China; 4 Department of Medicine, School of Medicine, University of California, San Diego, CA 92093, USA

**Keywords:** Small intestine, Kv channel subtypes, Glucose absorption, Anion secretion, Diabetes mellitus

## Abstract

Although K^+^ channels are important in mediating the driving force for colonic ion transport, their role in small intestinal transport is poorly understood. To investigate this, small intestinal short circuit currents (*I_sc_*) and HCO_3_^−^ secretion were measured in mice, and intracellular pH (pH_i_) was measured in small intestinal epithelial SCBN cells. The expression and location of Kv subtypes were verified by RT-PCR, western blotting and immunohistochemistry. Diabetic mice were also used to investigate the role of Kv subtypes in regulating intestinal glucose absorption. We found that K_V_7.1 is not involved in duodenal ion transport, while K_Ca_3.1 selectively regulates duodenal *I_sc_* and HCO_3_^−^ secretion in a Ca^2+^-mediated but not cAMP-mediated manner. Blockade of K_Ca_3.1 increased the rate of HCO_3_^−^ fluxes via cystic fibrosis transmembrane conductance regulator (CFTR) channels in SCBN cells. Jejunal *I_sc_* was significantly stimulated by glucose, but markedly inhibited by 4-aminopyridine (4-AP) and tetraethylammonium (TEA). Moreover, both Kv1.1 and Kv1.3 were expressed in jejunal mucosae. Finally, 4-AP significantly attenuated weight gain of normal and diabetic mice, and both 4-AP and TEA significantly lowered blood glucose of diabetic mice. This study not only examines the contribution of various K^+^ channel subtypes to small intestinal epithelial ion transport and glucose absorption, but also proposes a novel concept for developing specific K^+^ channel blockers to reduce weight gain and lower blood glucose in diabetes mellitus.

## Abstract (precis)

**Summary:** This study examines the different functions of various K+ channel subtypes in small intestinal epithelial, which proposes a novel concept for the development of weight-loss drugs and hypoglycemic drugs.

## INTRODUCTION

The small intestinal epithelium has two important physiological functions: the absorption of nutrients and the secretion of ions. Both processes are associated with electrogenic ion transport across the plasma membrane of intestinal epithelial cells (IECs) (Heitzmann and Warth, 2008). The absorption of most nutrients in the small intestine, such as glucose (Wright et al., 1997), amino acids (Stevens, 1992) and long-chain free fatty acids (Berk and Stump, 1999; Berk et al., 1997; Stremmel, 1988), is usually associated with electrogenic activity. For example, glucose uptake in the jejunum is driven by the transmembrane Na^+^ gradient and the membrane potential. The major route for entry of dietary glucose from the jejunal lumen into enterocytes is Na^+^/glucose cotransport via Na^+^/glucose co-transporter 1 (SGLT1) (Gorboulev et al., 2012). Although the membrane potential of IECs is important in regulating the activity of SGLT1, the membrane potential itself is maintained by plasma membrane ion channels and transporters. While the Na^+^/glucose cotransporter has been extensively studied in jejunal glucose absorption, little is currently known regarding their regulation by plasma membrane ion channels, such as K^+^ channels in IECs.

The small intestinal epithelium also secretes and reabsorbs electrolytes such as Cl^−^ and HCO_3_^−^. Intestinal Cl^−^ secretion plays an important role in regulating the amount of fluid in the intestinal lumen, while HCO_3_^−^ secretion in the upper GI tract is crucial to protecting the vulnerable epithelium against gastric acid and pepsin (Barrett, 1997; Bedine, 2000). Indeed, the importance of duodenal mucosal HCO_3_^−^ secretion can be readily demonstrated in patients with duodenal ulcers, in whom acid-stimulated HCO_3_^−^ secretion is attenuated by 41% compared to healthy subjects (Allen and Flemström, 2005; Allen et al., 1993; Flemström and Isenberg, 2001; Isenberg et al., 1987). It has become widely accepted that epithelial transport relies on the membrane potential, which provides the driving force necessary for movement of nutrients and electrolytes across the plasma membrane. Growing evidence indicates that epithelial K^+^ channels play an important role in maintaining this driving force as well as stabilizing the membrane potential (Warth, 2003).

K^+^ channels are ubiquitously expressed in almost all excitable or non-excitable cells, including IECs. There are several subtypes of K^+^ channels already described in IECs, including Kv1.1 (KCNA1), Kv1.3 (KCNA3), KCa3.1 (KCNN4) and Kv7.1 (KCNQ1) (Heitzmann and Warth, 2008). These channels are implicated in a variety of cellular functions, such as ion transport, volume regulation, cell migration, wound healing, proliferation, apoptosis and carcinogenesis (Heitzmann and Warth, 2008). Although it is known that K^+^ channels are important in stabilizing the membrane potential and mediating the driving force for electrogenic ion transport in colonic epithelial cells, their role in regulating epithelial transport remains to be elucidated in the small intestine. Therefore, the aims of the present study are to identify if K^+^ channels subtypes, Kv1.1, Kv1.3, KCa3.1 and Kv7.1 are functionally expressed in the small intestinal epithelium; and if so, whether they are involved in controlling physiological functions. Here, we describe various roles for K^+^ channel subtypes in small intestinal epithelial transport, specifically in regulating duodenal anion secretion and jejunal glucose absorption. Our study also provides incentive for developing specific blockers for K^+^ channel subtypes as novel therapeutic agents to modulate intestinal epithelial secretion, reduce weight gain and improve glycemic control in diabetes mellitus.

## RESULTS

### Role of K_V_7.1 (KCNQ1) in duodenal anion secretion

**Fig. 1.**
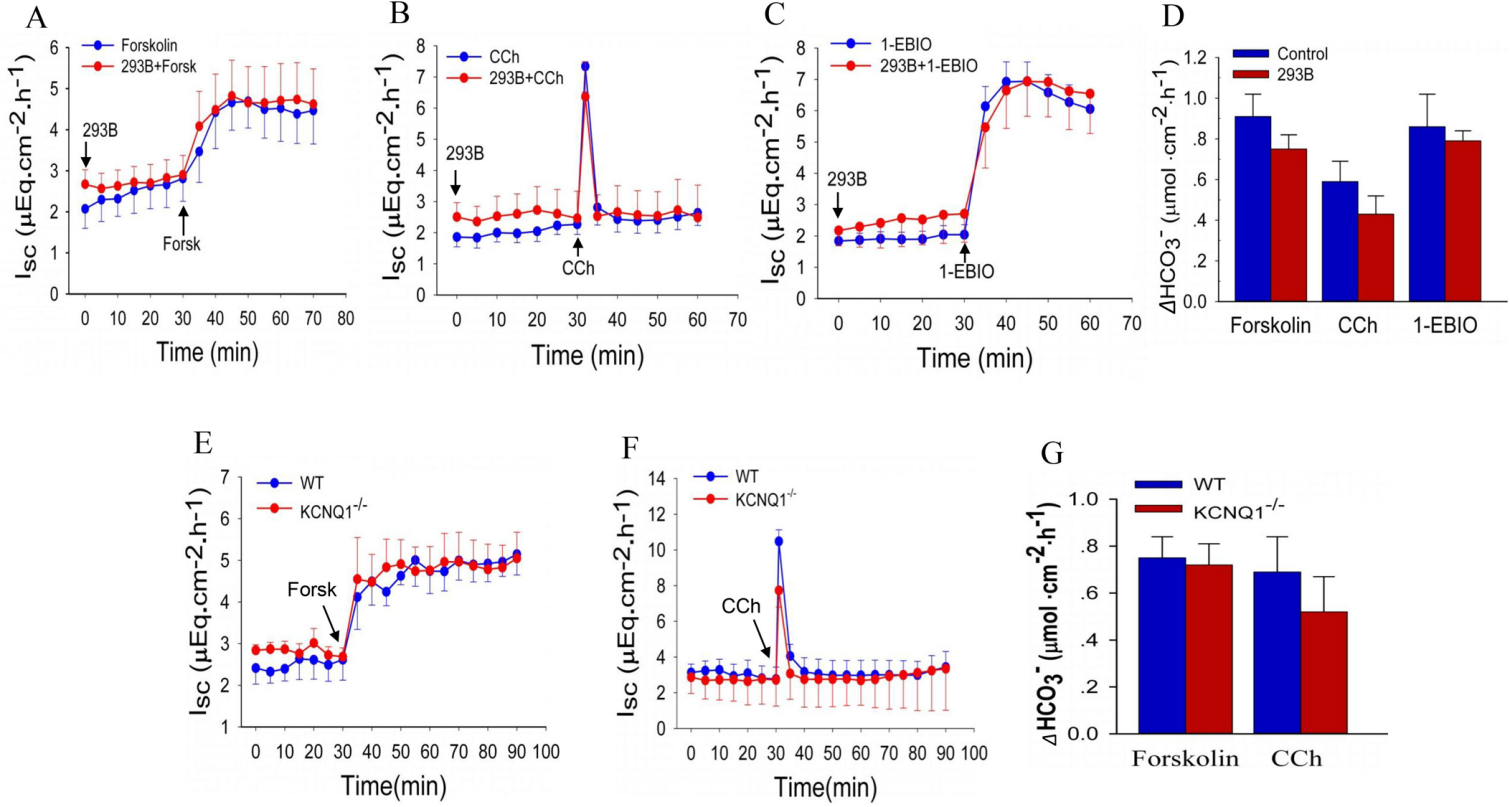
**No involvement of Kv7.1 (KCNQ1) subtype in duodenal ion transport in mice.** Forskolin (A), CCh (B) and 1-EBIO (C) stimulated duodenal *I*_sc_ and net peak HCO_3_^−^ secretion (D) in the absence or the presence of 293B in C57BL/6J WT mice. 293B (10 μM) and 1-EBIO (1 mM) were added to both sides, but forskolin (10 μM) and CCh (100 μM) were added to the serosal sides at the times indicated. Forskolin- (E) and CCh- (F) stimulated mucosal *I*_sc_ and net peak HCO_3_^−^ secretion (G) in K_V_7.1^−/−^ mice and WT mice. Forskolin (10 μM) and CCh (100 μM) were added to the serosal sides at the times indicated. Values are means±s.e.m.; *n*=5–7 tissues in each series; Student's *t*-test, no statistical significances were identified between each group.

Since K_V_7.1 channels have been shown to play roles in regulating the secretion of gastric acid and jejunal Cl^−^ (Dong et al., 2006), we began by testing whether K_V_7.1 channels were functionally involved in duodenal anion secretion. We chose three different agents for pharmacological stimulation of epithelial anion secretion: (1) forskolin, through triggering the cAMP pathway; (2) carbachol (CCh), by mobilizing intracellular Ca^2+^; (3) 1-ethyl-2-benzimidazolinone (1-EBIO), which activates the K_Ca_ channel by increasing its Ca^2+^ sensitivity. When introduced to the serosal side of duodenal tissue from C57BL/6J mice, forskolin (10 μM), CCh (100 μM), and 1-EBIO (1 mM) all induced significant intestinal short circuit current (*I_sc_*) and HCO_3_^−^ secretion (Fig. 1A–D). The net peak HCO_3_^−^ secretion (Fig. 1D) was calculated as the peak HCO_3_^−^ secretion at 10 min minus the basal value. Addition of a selective K_V_7.1 blocker, chromanol 293B (10 μM), to both sides did not alter the time courses of these responses. Furthermore, we did not find any difference in forskolin- and CCh-induced duodenal *I_sc_* (Fig. 1E,F) and net peak HCO_3_^−^ secretion (Fig. 1G) between K_V_7.1^−/−^ mice and wild-type mice. Therefore, consistent with Liao's previous report (Buresi et al., 2002), we confirmed that K_V_7.1 channels are not involved in Cl^−^ and HCO_3_^−^ secretion mediated by cAMP and Ca^2+^ signaling in the duodenum.

### Specific role of K_Ca_3.1 (KCNN4) channels in Ca^2+^-mediated duodenal anion secretion

**Fig. 2.**
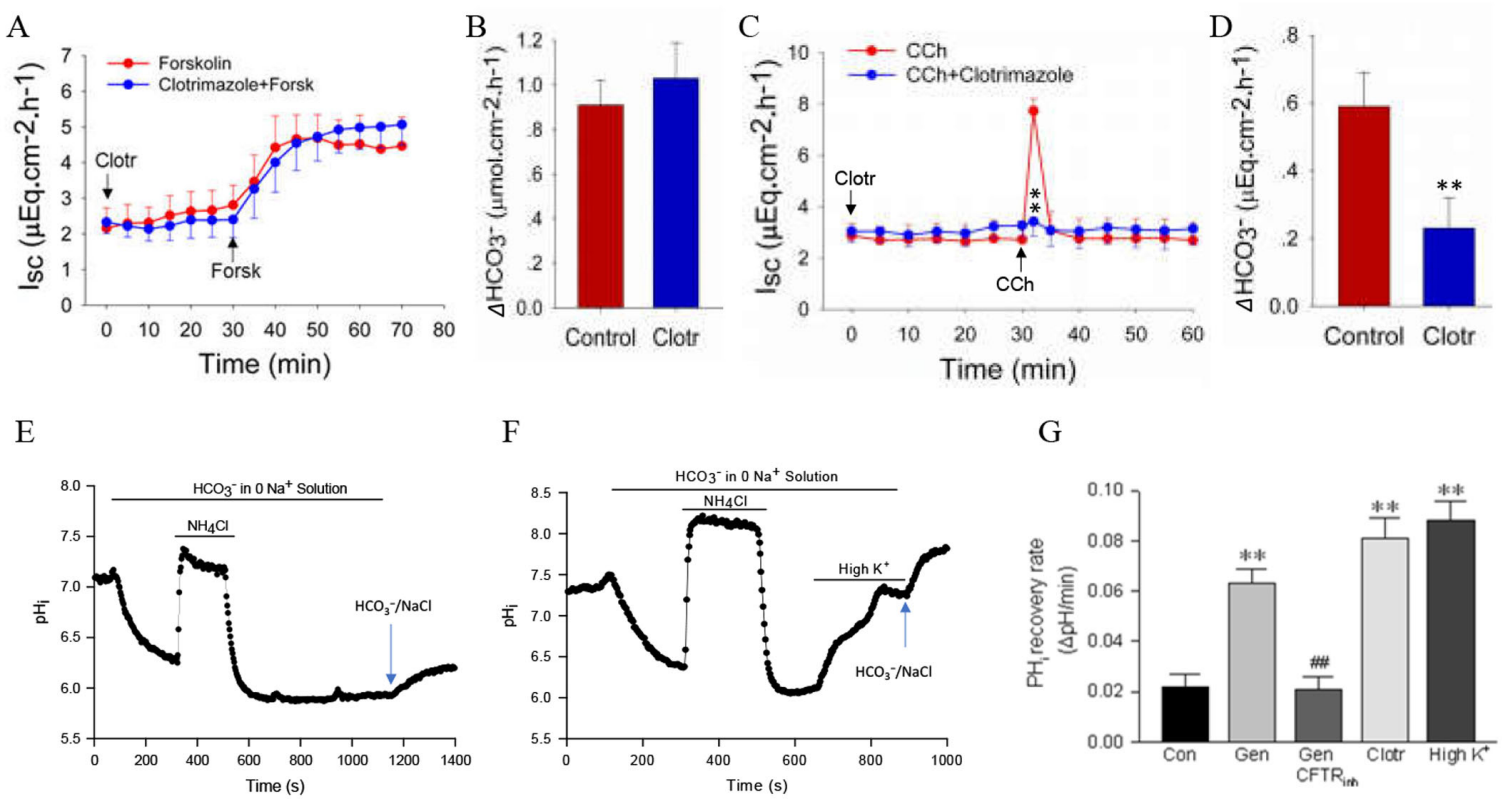
**Important role of Ca^2+^-mediated K_Ca_3.1 (KCNN4) subtype in duodenal ion transports.** (A,B) Forskolin-stimulated duodenal *I*_sc_ and net peak HCO_3_^−^ secretion in the presence of clotrimazole in WT mice. (C,D) CCh-stimulated duodenal *I*_sc_ and net peak HCO_3_^−^ secretion in the presence of clotrimazole in K_V_7.1^−/−^ mice. Clotrimazole (30 μM) was added to both sides, but forskolin (10 μM) and CCh (100 μM) were added to the serosal sides at the time indicated. **P<0.01 versus control; *n*=5–6 tissues in each series. (E) Control time course of pH*_i_* changes induced by NH_4_Cl (30 mM) in Na^+^-free/HCO_3_^−^ solution, in which pH*_i_* first increases and then decreases after washout. The cells remained acidic, with relatively stable pH*_i_*, which began to recover after addback of Na^+^/HCO_3_^−^ solution (the last peak). (F) High K^+^-induced HCO_3_^−^ fluxes through CFTR channels. The time course of pH*_i_* changes in cells was similar to the control in E except high K^+^ was added to the cells acidified in HCO_3_^−^/0Na^+^ solution as indicated. (G) Summary data showing the effects of genistein (Gen*,* 30 μM), CFTR_inh_-173 (10 μM), clotrimazole (Clotr, 30 μM), and high K^+^ (80 mM) on HCO_3_^−^ fluxes in SCBN cells. Student's *t*-test, **P<0.01 versus control (Con), and ^##^*P*<0.01 versus Gen, *n*=40–50 cells in each series.

Although we previously demonstrated the important role of K_Ca_3.1 channels in regulating duodenal Cl^−^ and HCO_3_^−^ secretion in mice (Cheng, 2012), it is unclear whether K_Ca_3.1 channels are specifically activated through the Ca^2+^ pathway for this process. Therefore, both cAMP and Ca^2+^ pathways were examined in the present study. Selective blockade of K_Ca_3.1 with clotrimazole (30 μM) did not alter the time course of forskolin-induced duodenal *I_sc_* and net peak HCO_3_^−^ secretion in wild-type mice (Fig. 2A,B), excluding the non-selectivity of clotrimazole for the cAMP signaling pathway. To confirm this notion, we further examined the effect of clotrimazole on CCh-induced duodenal *I_sc_* and net peak HCO_3_^−^ secretion in K_V_7.1^−/−^ mice, to exclude the possible involvement of K_V_7.1 channels in duodenal Cl^−^ and HCO_3_^−^ secretion. We found that clotrimazole (30 μM) significantly attenuated the time course of CCh-induced duodenal *I_sc_* and net peak HCO_3_^−^ secretion in these mice (Fig. 2C,D). By combining selective pharmacological blockade and genetic knockout mice, we confirm that K_Ca_3.1 channels are involved in regulating Ca^2+^- but not cAMP-mediated duodenal anion secretion.

Since the cystic fibrosis transmembrane conductance regulator (CFTR) channel has been characterized in SCBN cells and they are often used to study epithelial anion secretion (Buresi et al., 2001, 2005; Vallon et al., 2005), we chose this cell line to test the role of CFTR channels in HCO_3_^−^ entry. SCBN cells were treated with NH_4_Cl in Na^+^-free/HCO_3_^−^ solution, which initially caused an increase in pH_i_ due to entry of the weak base NH_3_, followed by a decrease in pH_i_ when NH_4_^+^ was washed from the extracellular solution. Subsequently, pH_i_ gradually recovered due to likely entry of HCO_3_^−^ through CFTR channels, and addback of Na^+^/HCO_3_^−^ solution resulted in further recovery of pH_i_ through both CFTR and Na^+^-dependent mechanisms of HCO_3_^−^ entry (Fig. 2E). The recovery rate of pH_i_ was calculated to represent HCO_3_^−^ entry via CFTR channels after NH_4_^+^ washout with Na^+^-free/HCO_3_^−^ solution. Indeed, the pH_i_ recovery rate was increased by the CFTR activator genistein (30 μM) (Tuo et al., 2009) and significantly inhibited by the CFTR blocker CFTRinh-172 (10 μM) (Fig. 2G). These data provide strong evidence for the important role of CFTR channels in HCO_3_^−^ entry in SCBN cells (Buresi et al., 2001). When high K^+^ (80 mM) was added to induce membrane depolarization in SCBN cells that were acidified in Na^+^-free/HCO_3_^−^ solution, a quick initial pH_i_ recovery was observed, with further recovery after addback of Na^+^/HCO_3_^−^ solution (Fig. 2F,G). Similar experiments were performed with clotrimazole (30 μM), which also increased the pH_i_ recovery rate in Na^+^-free/HCO_3_^−^ solution (Fig. 2G). These results not only provide further evidence to support our previous finding that K_Ca_3.1 channels are important for HCO_3_^−^ secretion, but also elucidate the underlying mechanisms of K_Ca_3.1-mediated HCO_3_^−^ entry through CFTR channels in SCBN cells (Buresi et al., 2001).

### Glucose induces jejunal *I_sc_* via the Na^+^/glucose co-transporter

**Fig. 3.**
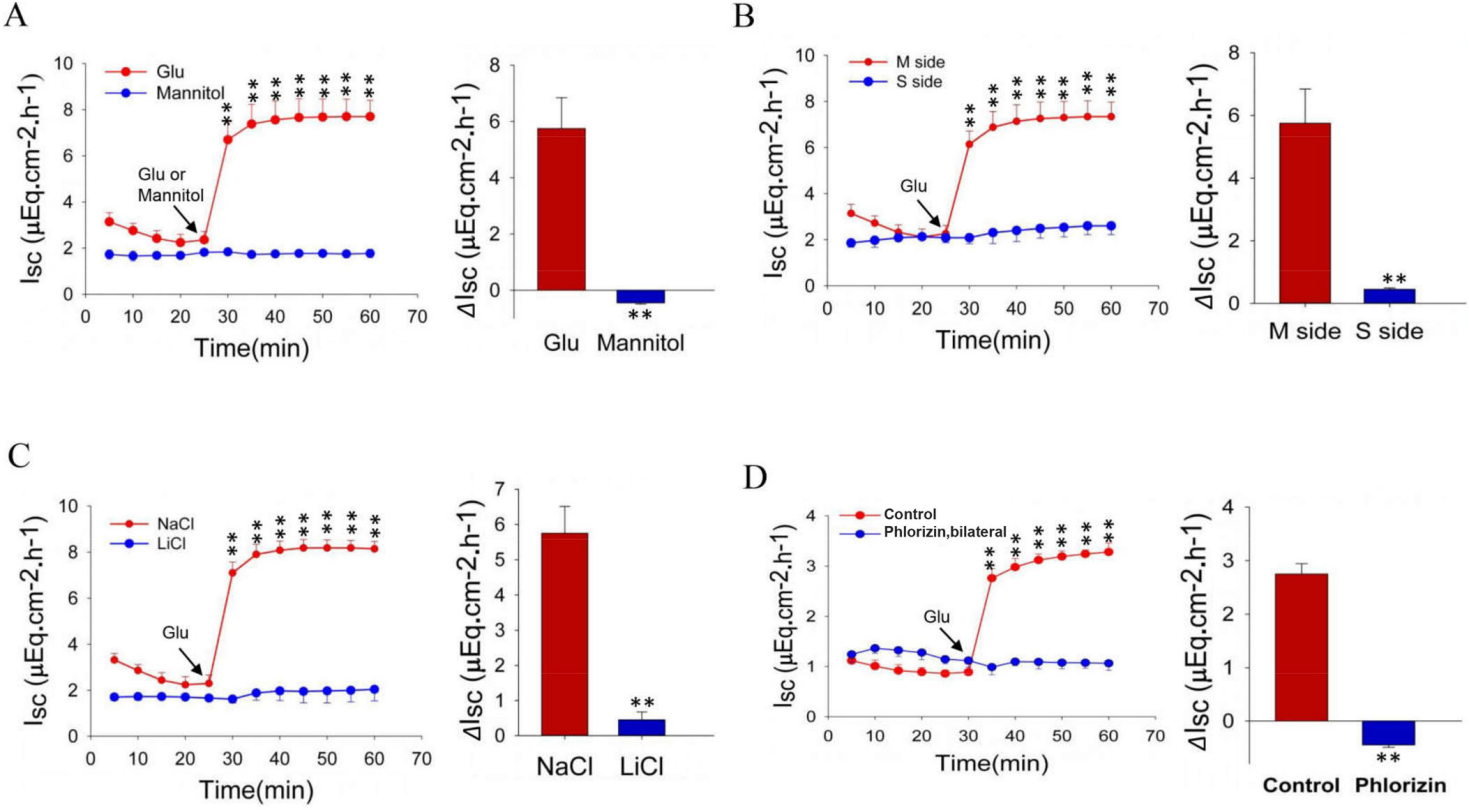
**The time courses and net peaks of jejunal glucose absorption under various conditions.** (A) Comparison of jejunal *I_sc_* induced by mucosal addition of glucose (Glu) or mannitol in the presence of extracellular Na^+^ (140 mM). (B) Comparison of jejunal *I_sc_* induced by mucosal (M side) or serosal (S side) addition of glucose in the presence of extracellular Na^+^. (C) Comparison of jejunal *I_sc_* induced by M side addition of glucose in the presence of NaCl or LiCl (the absence of extracellular Na^+^). (D) Inhibitory effect of phlorizin (1 mM) on jejunal *I_sc_* induced by mucosal addition of glucose in the presence of extracellular Na^+^. Glucose or mannitol (25 mM for both) was added at the time indicated. These tests were conducted in WT mice. Values are means±s.e.m.; Student's *t*-test, **P<0.01 versus control, *n*=4–5 tissues in each series.

Since it is well known that jejunal active glucose absorption is through mucosal SGLT1, we conducted Ussing chamber experiments to record jejunal *I_sc_* in wild-type mice. First, when glucose (25 mM) was added to the mucosal side of jejunal tissue, it induced marked *I_sc_* while mannitol (25 mM), a similar organic compound, also derived from sugar, did not (Fig. 3A). Second, mucosal addition of glucose induced marked *I_sc_*, but not serosal addition (Fig. 3B). Third, mucosal addition of glucose induced marked *I_sc_* in the presence of NaCl, but not in the presence of LiCl (Na^+^-free substitute) (Fig. 3C). Finally, glucose-induced *I_sc_* in the presence of NaCl was abolished by phlorizin (1 mM), a specific inhibitor of SGLT1 (Fig. 3D). Together, these results strongly suggest that jejunal mucosal Na^+^/glucose co-transporter (i.e. SGLT1) mediates this glucose-induced intestinal *I_sc_*.

### Roles of K^+^ channel subtypes in the regulation of jejunal glucose absorption

**Fig. 4.**
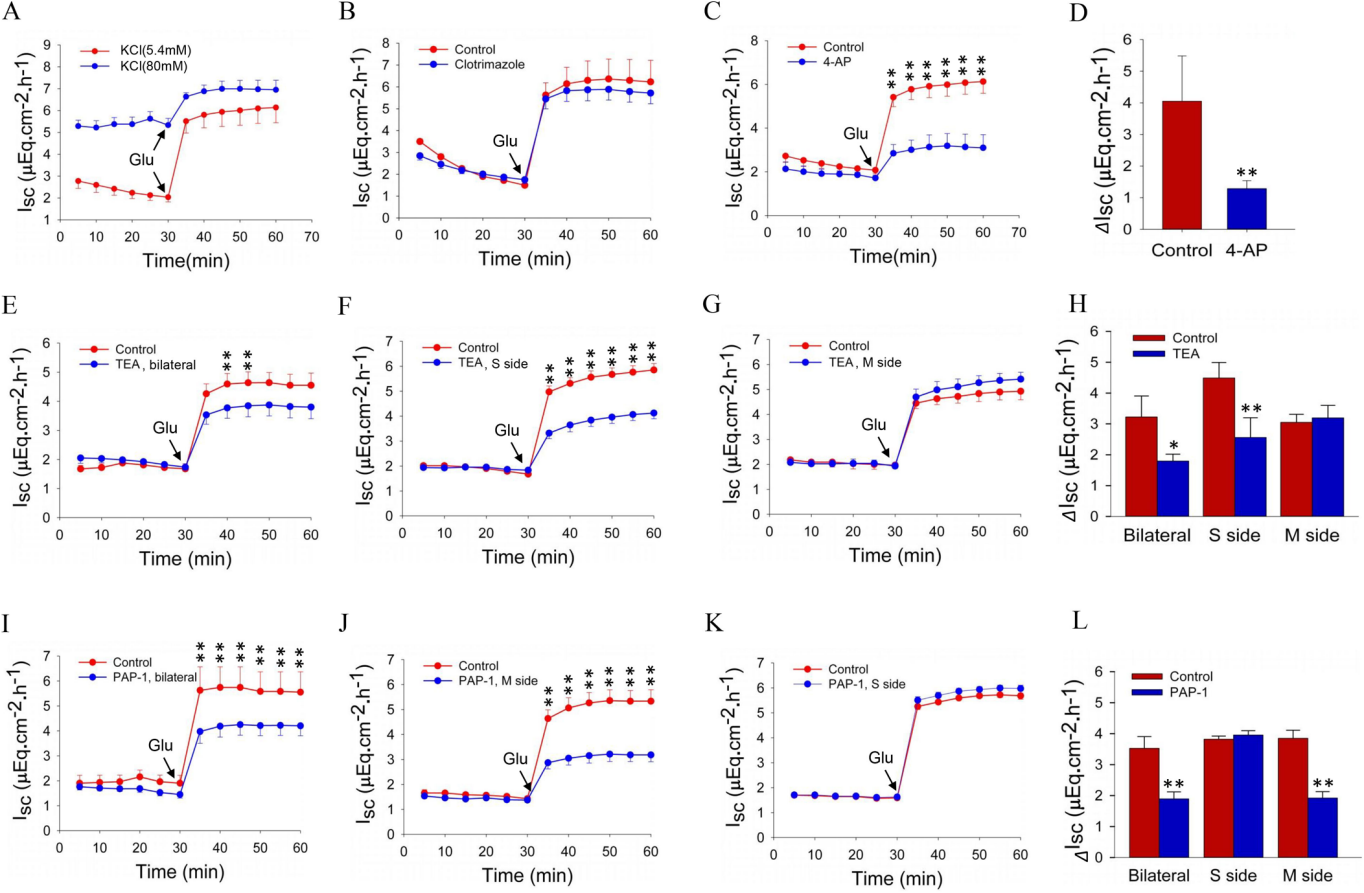
**Effects of selective blockers for Kv channel subtypes on time courses and net peaks of jejunal glucose absorption.** (A,B) Glucose-induced jejunal *I_sc_* after bilateral addition of high K^+^ (80 mM) or clotrimazole (30 μM). (C,D) Effect of addition of 4-AP (1 mM) to both sides on time courses and net peaks of jejunal glucose absorption. (E–G) Glucose-induced *I_sc_* after bilateral, serosal or mucosal addition of TEA (0.5 mM). (H) Effect of TEA on net peak of jejunal glucose absorption after bilateral, serosal or mucosal addition. (I–K) Glucose-induced jejunal *I_sc_* after bilateral, mucosal or serosal addition of PAP-1 (0.5 μM). (L) Effect of PAP-1 on net peak of jejunal glucose absorption after bilateral, mucosal or serosal addition. Glucose (25 mM) was added at the time indicated after pretreatment with the selective Kv subtype blockers. Values are means±s.e.m.; Student's *t*-test, *P<0.05, **P<0.01 versus control, *n*=5–7 tissues in each series.

To test the general roles of membrane potential and K^+^ channels in the regulation of nutrient absorption, high K^+^ (80 mM) was added bilaterally to Ussing chambers to inhibit all plasma membrane K^+^ channels. As shown in Fig. 4A, high K^+^ not only raised the baseline jejunal *I_sc_* but also inhibited the glucose-induced *I_sc_*, indicating the critical role of K^+^ channels in the regulation of nutrient absorption. Since K_Ca_3.1 channels are important in intestinal *I_sc_* and HCO_3_^−^ secretion, we tested if K_Ca_3.1 plays a role in regulating jejunal glucose absorption; however, clotrimazole (30 μM) did not alter glucose-induced jejunal *I_sc_* (Fig. 4B). We then tested 4-aminopyridine (4-AP) (1 mM), a broad-spectrum blocker of Kv channels (Castle et al., 2003), and found it abolished *I_sc_* (Fig. 4C,D). Thus, we focused on Kv channels and their potential role in the regulation of jejunal glucose absorption, using the selective K_V_1.1 inhibitor tetraethylammonium (TEA) (Castle et al., 2003). We found that glucose-induced jejunal *I_sc_* is significantly attenuated with bilateral addition of TEA (0.5 mM) (Fig. 4E), and specifically only when added to the serosal side, but not the mucosal side (Fig. 4F,G). This suggests the functional expression of K_V_1.1 is polarized (Fig. 4H), and these findings overall reveal a role for serosal K_V­_1.1 channels in the regulation of jejunal glucose absorption.

Next, we shifted our focus to Kv1.3 channels. As shown in Fig. 4I, bilateral addition of 5-(4-phenoxybutoxy) psoralen (PAP-1) (0.1 μM) significantly attenuated glucose-induced *I_sc_*. To determine if functional expression of Kv1.3 is polarized, PAP-1 was selectively added to either side of epithelial cells, and glucose-induced *I_sc_* was only attenuated when PAP-1 was added to the mucosal side rather than the serosal side (Fig. 4J,K), in contrast to TEA. Fig. 4L summarizes the effect of PAP-1 on the net peak jejunal glucose absorption after either mucosal, serosal or bilateral addition. These findings highlight the potential role of mucosal Kv1.3 channels in the regulation of jejunal glucose absorption.

### Expression and location of Kv1.1 and Kv1.3 channels in jejunal epithelium

**Fig. 5.**
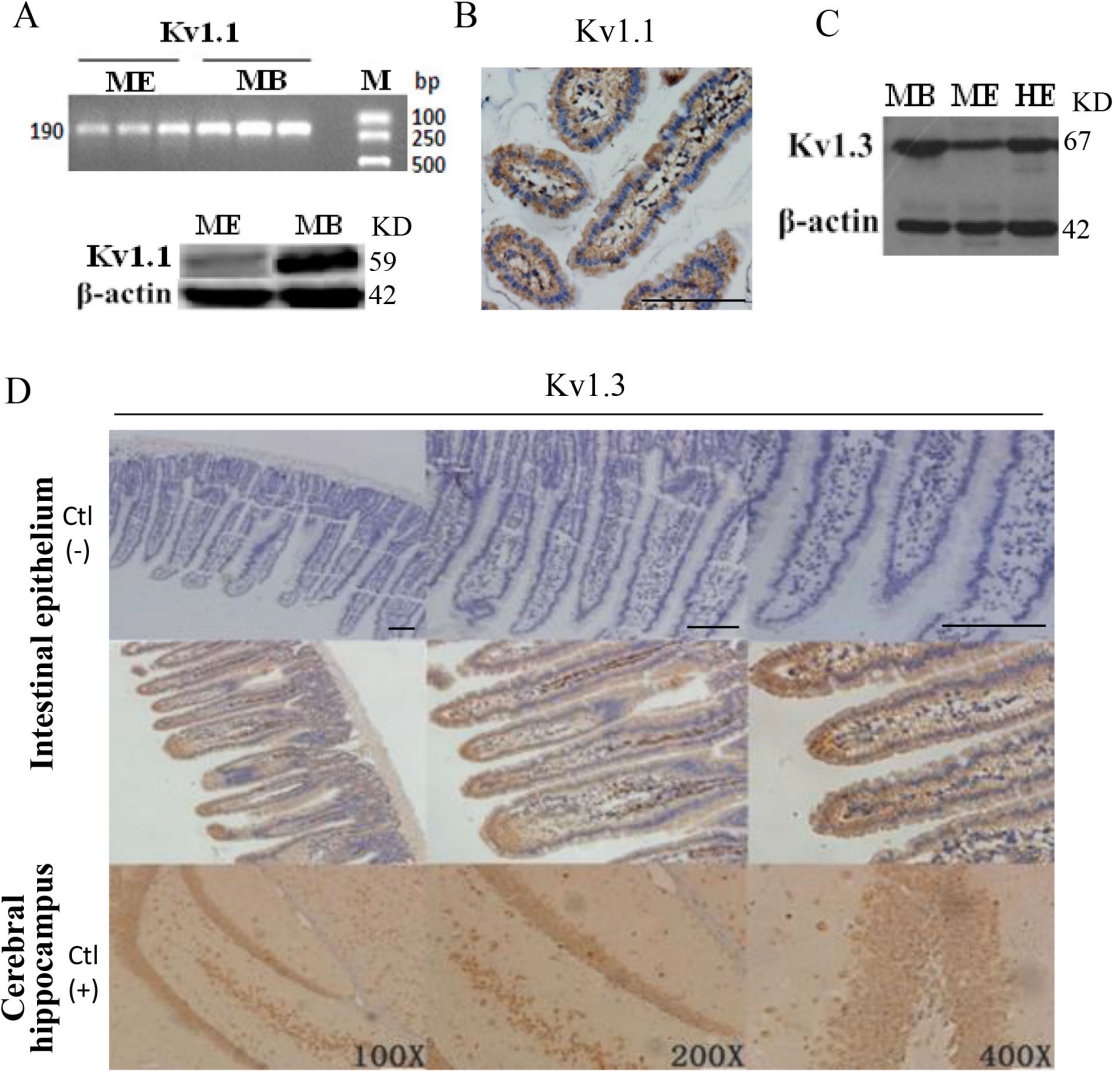
**Expression and location of Kv1.1 and Kv1.3 in jejunal epithelium.** (A) Kv1.1 expression of mRNA (upper) and protein (lower) in mouse jejunal epithelium (ME) and mouse brain (MB) as a positive control. M, marker; KD, molecular weight. (B) Expression and localization of Kv1.1 protein in epithelial cells of mouse jejuna. (C) Protein expression of Kv1.3 in MB as a positive control, in ME and human jejunal epithelium (HE). (D) Expression and localization of Kv1.3 protein in epithelial cells of mouse jejunum and mouse cerebral hippocampus. Upper panels: immunohistochemistry staining of mouse jejunal epithelium without primary antibody as a negative control. Middle panels: immunohistochemistry staining of the epithelial cells in mouse jejunal epithelium with primary anti-Kv1.3 antibody. Lower panels: immunohistochemistry staining of the hippocampal neurons in mouse brain with primary anti-Kv1.3 antibody as a positive control. These are representative of three independent experiments with similar results. Scale bars: 200 μm, 100 μm, 50 μm.

Although both Kv1.1 and Kv1.3 channels have been identified in the brain and other tissues, their expression and localization are not well characterized in the jejunal intestinal epithelium. We used PCR analysis to detect mRNA expression of Kv1.1 transcripts in mouse jejunal epithelium (ME), while mouse brain (MB) served as a positive control (Fig. 5A). Western blot analysis was performed and detected the protein expression of Kv1.1 in both ME and MB by anti-K_V_1.1 antibody (Fig. 5A). Immunohistochemistry analysis further identified the expression and location of Kv1.1 channels in epithelial cells (Fig. 5B). Similarly, Kv1.3 protein expression was also detected by western blot analysis in MB, ME and human jejunal epithelium (HE) (Fig. 5C), and immunohistochemistry analysis identified the expression and location of Kv1.3 channels in mouse epithelial cells and mouse cerebral hippocampal neurons (Fig. 5D). No staining was observed on mouse epithelial cells without primary anti-Kv1.1 and anti-Kv1.3 antibodies (Fig. 5D), suggesting the observed results are specific to K_V_1.1 and K_V_1.3 channels.

### Roles of Kv channel subtypes in the regulation of body weight and blood glucose in mice

**Fig. 6.**
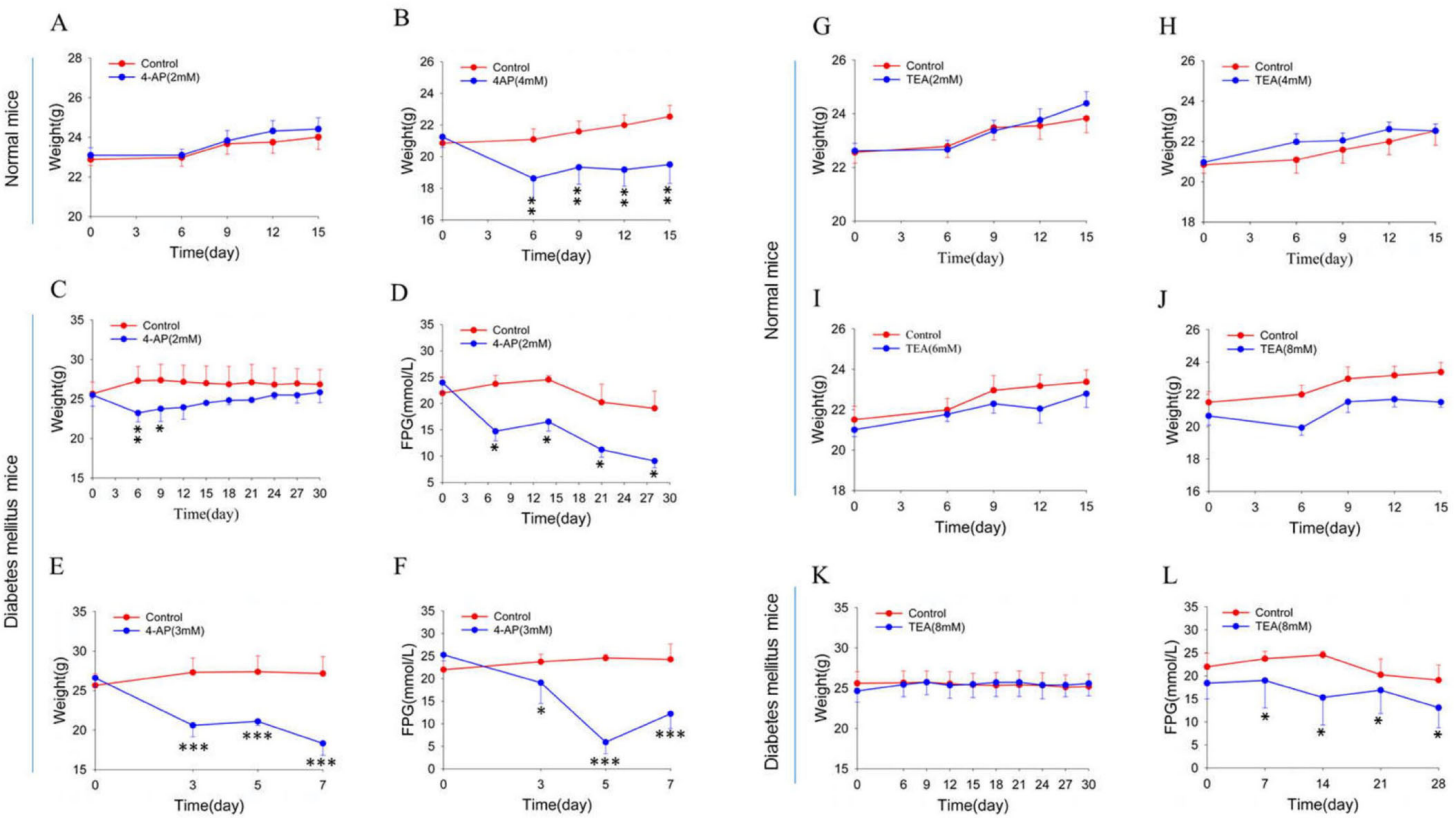
**Effects of 4-AP and TEA on time courses of body weight in normal mice and measurement of body weight and blood glucose in diabetic mice.** (A,B) Effect of 4-AP (2 and 4 mM) on body weight in normal mice. (C,D) Effect of 4-AP (2 mM) on body weight and fasting plasma glucose (FPG) in diabetic mice. (E,F) Effect of 4-AP (3 mM) on body weight and FPG in diabetic mice. (G–J) Effect of TEA (2–8 mM) on body weight in normal mice. (K,L) Effect of TEA (8 mM) on body weight and FPG in diabetic mice. Mice were given drinking water only (control) or drinking water containing different concentrations of 4-AP or TEA. Values are means±s.e.m.; Student's *t*-test, *P<0.05, **P<0.01, ***P<0.001, *n*=6–7 mice in each series.

Since our *in vitro* Ussing chamber experiments showed that Kv channels are important in the regulation of intestinal glucose absorption, we further examined their roles *in vivo*. First, body weight in wild-type mice were measured and compared between the control group given water only and the experimental groups given water containing 4-AP (2 and 4 mM) for 2 weeks. 4-AP at 4 mM significantly decreased body weight of wild-type mice by 20%, but no difference was observed at 2 mM (Fig. 6A,B). Second, in a mouse model of diabetes mellitus, 4-AP caused weight loss (Fig. 6C,E) and reduced blood glucose (Fig. 6D,F) in a dose-dependent manner. Interestingly, at 2–8 mM TEA did not significantly alter body weight in wild-type mice (Fig. 6G–J), but in diabetic mice, 8 mM TEA lowered blood glucose (Fig. 6L) without any effect on body weight (Fig. 6K). Taken together, our data suggest that Kv channels, especially the Kv1.1 subtype, may be involved in global control of body weight and blood glucose through regulating intestinal glucose absorption.

## DISCUSSION

Although the expression of K^+^ channel subtypes has been identified in GI epithelium, their physiologic function in the small intestine is poorly understood. In the present study, using a combination of pharmacologic blockers and genetic knockout mice for K^+^ channel subtypes we reveal that: (1) K_Ca_3.1 channels may specifically regulate Ca^2+^-mediated intestinal anion secretion, but not glucose absorption; (2) Kv1.1 and Kv1.3 channels may play important roles in regulating intestinal glucose absorption; (3) Kv channel subtypes may regulate body weight and blood glucose through modulation of glucose absorption in both normal and diabetic mice. Therefore, our results suggest that various subtypes of K^+^ channels play different roles in the regulation of small intestinal ion and glucose transport.

**Fig. 7.**
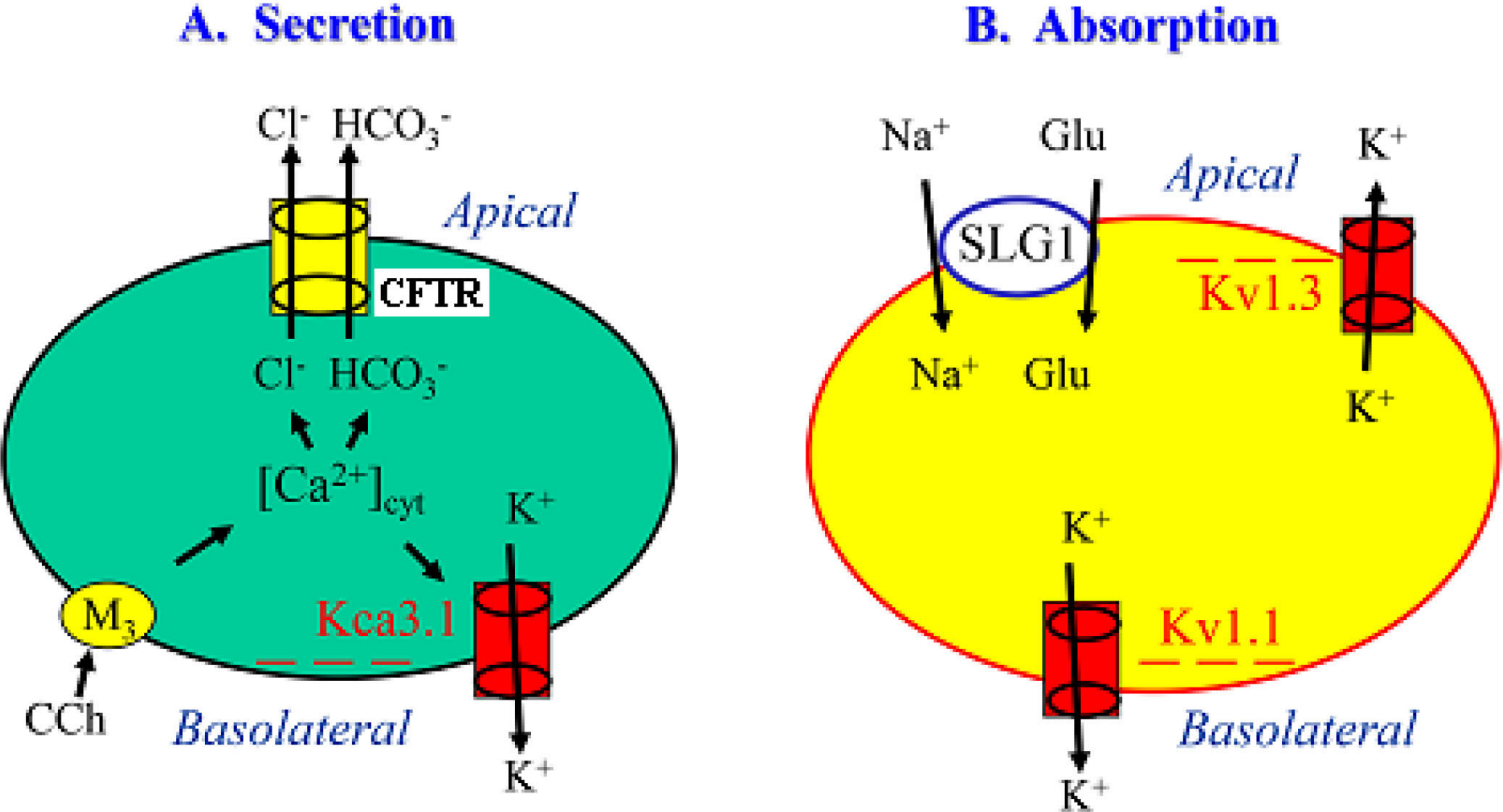
**Schematic diagram showing the different possible roles of various Kv channel subtypes in the regulation of ion and glucose transports through IECs.** (A) CCh activation of M3 receptors elicits [Ca^2+^]_cyt_ signaling in IECs, which activates the K_Ca_3.1 subtype on the basolateral side of IECs, leading to membrane hyperpolarization to provide a driving force for transepithelial Cl^−^ and HCO3^−^ flux through CFTR channels. (B) Glucose is absorbed from the intestinal lumen into IECs through SLGT1. This process is likely regulated by Kv1.3 channels functionally expressed on the apical side and by Kv1.1 channels functionally expressed on the basolateral side. Blockade of these Kv subtypes cause membrane depolarization, which would inhibit anion secretion and Na^+^-dependent glucose absorption by reducing the driving force for these electrogenic ion transports in IECs. − − −, membrane hyperpolarization.

It is generally accepted that K^+^ channels are important in stabilizing the membrane potential and mediating the driving force for electrogenic ion transport in colonic epithelium (Heitzmann and Warth, 2008). However, their expression and function in small intestinal epithelium are not well understood, and an even greater mystery is the involvement of different K^+^ channel subtypes in regulating ion and glucose transport. In a previous study (Cheng, 2012), we identified the expression of K_Ca_3.1 (KCNN4) in murine duodenal mucosae and characterized their role in regulating duodenal epithelial anion secretion. Although Matos et al. also confirmed the important role of K_Ca_3.1 in mouse colonic Cl^−^ secretion (Matos et al., 2007), the detailed mechanisms remain unclear for how K_Ca_3.1 channels regulate intestinal epithelial anion secretion, and almost nothing is known about K_Ca_3.1-mediated duodenal HCO_3_^−^ secretion. Therefore, we aimed to elucidate the underlying cellular mechanisms. It has been reported that K_Ca_3.1 activation induces membrane hyperpolarization (when *E_m_* becomes more negative inside of the plasma membrane) that likely provides the driving force for anion efflux out of colonic epithelial cells (Matos et al., 2007). Indeed, we measured HCO_3_^−^ flux through IECs and demonstrated for the first time that high K^+^ induced membrane depolarization (when *E_m_* becomes less negative inside of the plasma membrane) and increased the driving force for HCO_3_^−^ influx into IECs. Similarly, clotrimazole increased the driving force for HCO_3_^−^ influx into IECs likely via blockade of K_Ca_3.1 to induce membrane depolarization. Consistent with this, clotrimazole selectively inhibited Ca^2+^-mediated duodenal HCO_3_^−^ secretion *in vitro*. In the present study, we not only provided new evidence to support the notion that K_Ca_3.1 channels hold a critical role in regulating duodenal epithelial anion secretion, but also elucidated the underlying mechanism that Ca^2+^ activation of K_Ca_3.1 channels generates the driving force for HCO_3_^−^ secretion (Fig. 7A). Our findings agree with those from Flores's report that small intestine obtained from KCNN4 null mouse completely lack Cl^−^ secretion in response to Ca^2+^ mobilizing agonists, indicating the crucial role of K_Ca_3.1 in Ca^2+^-mediated epithelial ion transport (Flores et al., 2007). Thus, K_Ca_3.1 may represent a novel pharmacological target that could be exploited to manipulate small intestinal anion secretion in general and to specifically augment HCO_3_^−^ secretion to protect duodenal mucosa against acid-induced injury.

We also tested the possible involvement of other K^+^ channel subtypes, such as Kv7.1 (KCNQ1), since their roles in the regulation of intestinal ion transports have been controversial. Liao et al. previously reported that Kv7.1 channels were not essential for activating colonic epithelial Cl^−^ secretion (Buresi et al., 2002), but Matos et al. found that Kv7.1 channels drove colonic Cl^−^ secretion (Matos et al., 2007). However, only pharmacological blockers of Kv7.1 were used in those studies. By combining selective pharmacological blockers and K_V_7.1 knockout mice, we not only excluded the involvement of Kv7.1 in small intestinal anion secretion, but further confirmed the importance of K_Ca_3.1. Our results obtained in K_V_7.1 knockout mice differ from Vallon et al., who observed a decrease in forskolin-induced jejunal Cl^−^ secretion in K_V_7.1 knockout mice (Vallon et al., 2005). However, we consistently found no difference in forskolin-, 1-EBIO- and CCh-induced duodenal anion secretion using pharmacological blockers in both wild-type and knockout mice. The discrepancy between Vallon et al. and our study is likely due to the segment differences (duodenum versus jejunum), which requires further investigation.

Glucose is mainly imported into IECs through the SGLT1 located on the apical membrane in the small intestine (Wright et al., 1997; Stevens, 1992). In our Ussing chamber study, the glucose-induced intestinal *I_sc_* occurred specifically in the presence of both Na^+^ and glucose on the mucosal side of the small intestine, which was abolished by selective inhibition of SGLT1. This co-transporter is driven by the transmembrane Na^+^ gradient and the electrical potential difference (*E*_m_). Since the negative *E*_m_ inside IECs is a major driving force for movement of Na^+^ into the cell, membrane depolarization due to blockade of K^+^ channels may reduce the driving force for Na^+^, and subsequently inhibit Na^+^-dependent glucose absorption in IECs. K^+^ channels are presumably involved by repolarizing the cell membrane, which is critical in stabilizing the driving force for electrogenic Na^+^-coupled glucose transport. Indeed, we found that high K^+^ markedly inhibited glucose-induced intestinal *I_sc_*, indicating the negative *E*_m_ generated by K^+^ channels is essential for Na^+^-coupled glucose absorption. Interestingly, we show that although K_Ca_3.1 plays an essential role in Ca^2+^-mediated intestinal anion secretion, it is not involved in the regulation of intestinal glucose absorption. Consistent with this, we did not detect any glucose-induced changes in cytosolic Ca^2+^ concentrations in IECs (data not shown), excluding the role of these Ca^2+^-activated K_Ca_ channels in glucose absorption.

Although IECs express multiple Kv channel subtypes, such as Kv1.1, Kv1.3 and Kv7.1, their function in intestinal glucose transport are unclear except for the Kv7.1 subtype, which was previously reported to contribute to electrogenic Na^+^-coupled glucose transport in the jejunum (Dong et al., 2006). In the present study, we investigated the role of other Kv channel subtypes and identified the expression and localization of Kv1.1 and Kv1.3 channels in IECs of the small intestine in humans and mice. We demonstrated for the first time that both Kv1.1 and Kv1.3 channels were functionally expressed on the serosal and mucosal side, respectively, and that they are involved in the modulation of intestinal glucose absorption (Fig. 7B). Although the concentrations of K^+^ channel subtype blockers in the present study were higher than their EC_50_ values usually measured from cultured single cells, it is worth noting that we used primary intestinal tissues and whole-animal models. Under these more physiological conditions, it is much more complicated than cultured single cells, with varying factors such as higher cellular density and irregular drug access to the channels, etc. In view of this, the final verdict on Kv1.1 and Kv1.3 channels in regulating intestinal glucose absorption would require confirmation pending availability of their genetic knockout mice.

Kv1.1 and Kv1.3 subtypes are functionally expressed not only in IECs but also in smooth muscle cells of mesenteric arteries. The membrane depolarization in IECs induced by Kv channel blockers decreases the driving force that is required for Na^+^-driven glucose absorption in the small intestine. Moreover, membrane depolarization in smooth muscle cells would cause vasoconstriction of mesenteric arteries and limit the distribution of absorbed nutrients to other tissues for storage (McDaniel et al., 2001). Restriction of both nutrient absorption and distribution would reduce glucose intake and subsequent weight gain. Indeed, we found that 4-AP significantly attenuated the weight gain of wild-type and diabetic mice, and both 4-AP and TEA significantly lowered the blood glucose of diabetic mice. Therefore, Kv1.1 and Kv1.3 channels may play important roles in controlling glucose intake and weight gain. This study provides a new concept for developing specific blockers for K^+^ channel subtypes in the digestive system as novel therapeutic agents to reduce weight gain and to improve blood glucose control in diabetes mellitus.

## MATERIALS AND METHODS

### Ussing chamber experiments *in vitro*

This study was approved by the Committee on Investigations Involving Animal Subjects, Army Medical University. Experiments were performed with male 8–10-week-old male C57BL/6J mice and K_V_7.1 knockout mice (*Kv7.1*^−/−^), generated as previously described (Casimiro et al., 2001). Littermates (*Kv7.1*^+/+^) were used as wild-type controls.

Mice were anesthetized by i.p. injection of Hypnorm/Midazolam cocktail (25% Hypnorm plus 25% Midazolam) at a dose of 10 mg/kg. The duodenum and proximal jejunum were removed from C57BL/6J, *Kv7.1^−/−^*, or *Kv7.1*^+/+^ mice and immediately placed in ice-cold iso-osmolar mannitol with 10 μM indomethacin. The duodenal tissue from each animal was stripped of seromuscular layers, divided and mounted in three chambers (window area, 0.1 cm^2^). Experiments were performed under continuous short-circuited conditions (Voltage-Current Clamp, VCC 600; Physiologic Instruments, San Diego, USA). Luminal pH was maintained at 7.40 by continuous infusion of 5 mM HCl under automatic control of a pH-stat system (ETS 822; Radiometer America, Westlake, USA). The volume of HCl titrated was recorded in real time, then quantified as the steady-state rate of H^+^ equivalents required per hour for neutralization. The rate of HCO_3_^−^ secretion was calculated and normalized to tissue surface area (µmol cm^−2^ h^−1^). Measurements were taken at 5-min intervals and averaged for consecutive 5- or 10-min periods. The short-circuit current (*I_sc_*) was measured in microamperes and converted into µEq cm^−2^ h^−1^ (Dong et al., 2005). Basal parameters were initially recorded for the first 30 min, then with the addition of inhibitors for another 30 min, as dictated by the experimental design. Drugs were then added to the serosal side, the mucosal side or both, and electrophysiological parameters and bicarbonate secretion were measured for 60 min. As shown in our previous publications, in control experiments, addition of 10 µl vehicle (DMSO or distilled water) to both sides of the duodenal tissue in 3 ml chambers did not alter *I_sc_* or HCO_3_^−^ secretion, which were sustained during the 90 min experimental period.

The mucosal solution used in Ussing chamber experiments contained (in mM): 140 Na^+^, 5.4 K^+^, 1.2 Ca^2+^, 1.2 Mg^2+^, 120 Cl^−^, 25 gluconate and 10 mannitol. The serosal solution contained (in mM): 140 Na^+^, 5.4 K^+^, 1.2 Ca^2+^, 1.2 Mg^2+^, 120 Cl^−^, 25 HCO_3_^−^, 1.4 HPO, 2.4 H_2_PO_4_, 10 glucose and 0.01 indomethacin. The osmolality for both solutions were ∼285 mOsmol/kg H_2_O. In Na^+^-free solutions, Na^+^ was replaced with Li^+^*.*

### Epithelial cell culture

SCBN is a non-transformed duodenal epithelial crypt cell line of canine origin. As described previously (Pang et al., 1996; Buresi et al., 2001), cells in flasks were fed with fresh DMEM supplemented with 10% fetal bovine serum, l-glutamine, and streptomycin every 2–3 days (Buret and Lin, 2008). Cells of passages 23–33 were grown to confluence (∼5 days) in 75-cm^2^ flasks (Corning, USA), then replated onto 12-mm round coverslips (Warner Instruments Inc., Hamden, USA) and incubated for at least 24 h before use in pH*_i_* measurements.

### Measurement of HCO_3_^−^ fluxes in SCBN cells

SCBN cells were used for pH*_i_* measurements as previously described (Negulescu and Machen, 1990). Briefly, cells plated on coverslips were incubated with 2 μM 2′,7′-bis-(2-carboxyethyl)-5-(and-6)-carboxyfluorescein (BCECF), AM in physiological salt solution, for 30 min at room temperature then washed for 30 min. The ratio of BCECF fluorescence with excitation at 495 and 440 nm (*F*_495/440_) was captured using an intensified charge-coupled device camera and a MetaFluor imaging system. The NaCl/HCO_3_^−^ solutions contained (in mM): 120 NaCl, 25 NaHCO_3_, 2.5 K_2_HPO_4_, 1 MgSO_4_, 1 CaCl_2_ and 10 glucose, equilibrated with 5% CO_2_/95% O_2_ (pH 7.4). In Na^+^-free (Na^+^-free/HCO_3_^−^) solutions, Na^+^ was replaced with *N*-methyl-d-glucamine. The solutions were osmotically balanced with LiCl to ∼285 mOsmol/kg H_2_O and pH was adjusted to 7.4 with HCl.

### RT-PCR analysis

A RT-PCR analysis of mouse duodenal mucosae and brain was applied as previously described (Cheng, 2012). Briefly, total RNA from C57BL/6J mice duodenal mucosae and brain were isolated with TRIzol reagent (Invitrogen, Carlsbad, USA). Total RNA was converted into cDNA with reverse transcriptase (Takara, Japan). Primers were synthesized by Invitrogen. Each cDNA sample was prepared using AMV RT and random primers (Takara, Japan). Mice Kv1.1-specific sense and antisense primers (GenBank accession no. NM_010595.3) were 5′-AAGCTCTTACCCCTGCACTG-3′ and 5′-AACGGGTCTTAGCATTGGGG-3′. Mouse GAPDH sense and antisense primers as described by Sharkey, K. A. et al. were 5′-ACCACAGTCCATGCCATCAC-3′ and 5′-TCCACCACCCTGTTGCTGTA-3′ (Vallon et al., 2005). The samples were amplified in an automated thermal cycler (GeneAmp 2400, Applied Biosystems). The products were electrophoresed on a 1.5% agarose gel TAE buffer, stained with ethidium bromide (0.5 µg ml^−1^), then photographed under UV light.

### Western blot analysis

A western blot analysis of mouse duodenal mucosae and intestinal epithelial cells was conducted as previously described (Cheng, 2012). PVDF membranes (Millipore, Billerica, USA) with resolved proteins (50 µg) were incubated at 4°C overnight with anti-Kv1.1 (1:1000, sc-11184, Santa Cruz Biotechnology), anti-Kv1.3 (1:500, sc-398855, Santa Cruz Biotechnology), or anti-GAPDH antibodies (1:5000, Ambion, Austin, USA). After washing with PBS plus 1% Tween (PBST), the secondary antibody (rabbit anti-goat or anti-mouse, 1:1000, both from ZSGB-BIO, China) was applied to the membranes for 1 h at room temperature. Membranes were then treated with a chemiluminescent solution (Fivephoton Biochemicals, San Diego, USA) and captured on X-ray film. Densitometric analysis of the blots was performed using an AlphaImager digital imaging system (Alpha Innotech, San Leandro, USA).

### Immunohistochemistry

Immunohistochemistry was carried out as previously described (Liao et al., 2005). Briefly, dewaxed and rehydrated slides with small intestinal tissue from male C57BL/6J mice were blocked in goat serum for 1 h at room temperate, then incubated with anti-Kv1.1 (1:200) and anti-Kv1.3 (1:200) antibodies at 4°C overnight. The primary antibodies were detected with biotinylated rabbit anti-goat or goat anti-mouse IgG (1:5000, Vector Laboratories, Burlingame, USA) secondary antibodies for 1 h at room temperature. Immunoreactivity was detected using a horseradish peroxidase (3′-,3′-diaminobenzidine) kit (BioGenex, San Francisco, USA) followed by counterstaining with hematoxylin, dehydration and mounting.

### Determination of mouse body weight and streptozotocin-induced mouse model of diabetics

Male 8–10-week-old C57BL/6J mice were housed in an environmentally controlled facility with 12-h light/12-h dark cycles, and body weight was measured by scale every 3 days in the morning. Male C57BL/6J mice were fed with high-fat and high-glucose diets containing 18% lard oil, 20% sugar and 3% yolk. After 8 weeks, they were injected intraperitoneally with 35 mg/kg/day streptozotocin (STZ) for 7 consecutive days. Age-matched control mice received an equal volume of vehicle. After the seventh STZ injection, blood glucose levels were measured, and mice with blood glucose levels over 11.1 mmol/l were used for experiments.

### Chemicals

Carbachol (CCh, AchR activator), forskolin (Adenylate Cyclase activator), clotrimazole (KCa3.1 blocker), D-glucose and D-mannitol, TEA (Kv1.1 inhibitor), 4-AP (Kv blocker), PAP-1 (Kv1.3 inhibitor) and indomethacin were purchased from Sigma Chemical. 1-ethyl-2-benzimidazolinone (1-EBIO, Adenylate Cyclase activator) was from Tocris (Ellisville, USA). BCECF, AM was from Invitrogen.

### Statistical analysis

Results are expressed as mean±s.e.m. (standard error of the mean). Differences between means were considered to be statistically significant if *P*<0.05, using Student's *t*-test for paired or unpaired values, or analysis of variance, as appropriate.
